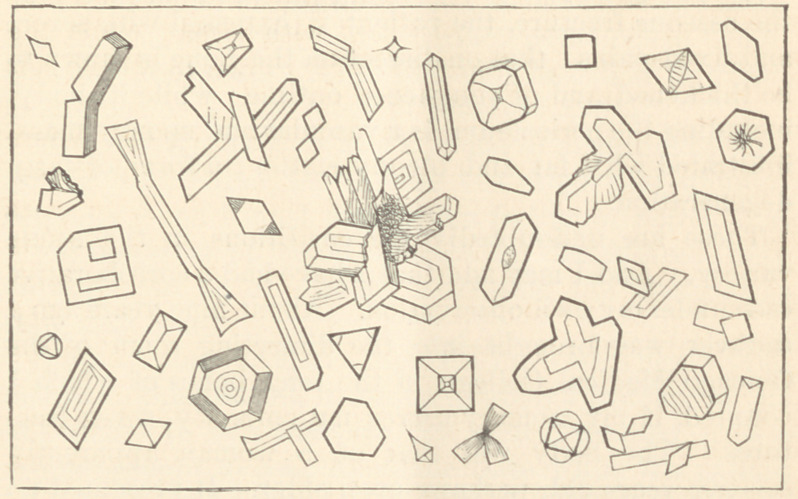# Rare Case of Fragilitas Ossium

**Published:** 1876-01

**Authors:** Wallace Blanchard

**Affiliations:** Chicago


### A RARE CASE OF FRAGILITAS OSSIUM.

By WALLACE BLANCHARD, M.D., Chicago.

In a general view of the subject, bone may be considered as made up of two proximate principles — the organic and the inorganic; upon the relative apportionment of which its strength depends.

A marked variation from a standard may be taken as a condition of disease; an excess of the organic matters constituting the rachitis so frequent in childhood and the osteo-malacia of adult life; while the opposite condition, of a preponderance of the inorganic or earthy materials or certain of them, gives us the fragility so often troublesome in the aged.

Fragility is very infrequent in early life, and when it does occur, I believe it may be of a different variety, pathologically, from the senile form. No attempt is made to indicate this in the medical literature at my command; surgical authors mainly agreeing with Grant that "fragility is pathologically the opposite condition to rickets," and giving but little further satisfaction as to the etiology and pathology of the disease.

Chassier gives a remarkable case in a child that survived its birth but twenty-four hours. Gross gives the case of a young man, "whose extremities were repeatedly broken by the most trivial accidents," and also a case of congenital fragility running through three generations, one of a family of children suffering five fractures.

The case here reported is that of Miss J——, aged twelve years and six months. At birth she was pronounced a healthy baby, but at her second month of life she suffered a fracture of the left femur, at or near the neck, and has demonstrated the brittleness of her osseous structure, every three or four months since that date, with astonishing regularity.

The mother's enumeration of fractures produced, is as follows: Right arm, 3; right fore-arm, 4; left fore-arm, 3; right thigh, 2; left thigh, 3; right leg, 14; left leg, 11; total, 40. To this I would add a well marked transverse fracture of the sternum, between the fourth and fifth ribs, which, though the mother and daughter are at variance as to the date of its occurrence, has certainly existed for a long time. This is the only fracture not occurring in the long bones of the limbs; and the left humerus is the only one of these long bones so far, which has escaped the accident.

In more than one-half the fractures of the fore-arms, both radius and ulna were broken simultaneously, and in all the fractures of the leg, the fibula gave way with the tibia. In fact, a solution of continuity in the long bones, has been repeated in the limbs of this child at least sixty-eight times.

We have but a slight suggestion in these fractures, of the laceration and contusion that accompany the fracture of a healthy bone, as well as of the inflammation and congestion that usually accompany it; consequently this patient has suffered but comparatively little pain.

The force required to produce a fracture in this case can hardly be termed violence; a step from the sofa to the carpet, or the most trivial slip or fall, being sufficient.

The process of union is tedious in the extreme. On removing the dressings at about the seventh or eighth week, the provisional bone uniting the fractured ends has about the firmness of the rubber in the pencil-mark eraser in common use; and two or three years are required to bring the union to any considerable firmness. Usually, however, the dressings have been removed at the second or third month, and the new union being of too pliable a material to resist the contracting power of the muscles, a gradual mal-position has followed; reaching its greatest consummation a little more than a year from the time of fracture, and in the year or two following, becoming comparatively firm in its new and vicious position. This has been repeated till the poor child is in truly a pitiable state of deformity.

In each case, the displacement has been of an angular variety, the callus being of sufficient strength at the time the splints were removed, to keep the fractured ends of the bone approximated.

In the cut made from a photograph, the seeming shortness of the thighs is largely due to their being nearly on a plane parallel to the camera. The lines running from the figures 1 and 2 indicate the elbows ; and those from 3 and 4 terminate at the lower edge of the patellæ. The distortions of the right fore-arm and thighs, are not as well shown as those of the left fore-arm and legs.

The family history gives no evidence of a scrofulous or other morbid taint, and both parents indignantly deny the possibility of any syphilitic infection. The mother says that from her first month of pregnancy up to the time of her delivery, she suffered constantly from sickness at the stomach ; being able at no time whatever to retain even the smallest amount of solid food, and that she lived almost entirely on essence and tea of peppermint slightly sweetened. One night during the second month of parturition, she suffered from urinary retention, accompanied by very severe pain, which was relieved by catheterization. For three months afterward her urine was voided with an intense burning sensation.

The girl's appetite is generally good, and her food is as nutritious and varied as could be desired; while the assimilating process seems to be as perfect in all the soft parts of the body, as could be expected.

The muscles are very well developed, except where they have become atrophied through a total want of use.

She has been at various times on such general tonics as iron, quinia, and strychnia, and for extended periods the vegetable acids have been exhibited, but so far with no perceptible effect on her fragile diathesis. She is very intelligent, and still retains considerable dexterity in the use of her hands and arms.

It may be stated that an inordinate quantity of earthy, especially phosphatic, matter, in the urine, is one of the diagnostic symptoms of this disease.

The microscopical examination of the urine of this little patient reveals a field of exceeding beauty. It is absolutely covered with crystalline forms. These are mainly of the phosphate of lime, with occasional octahedra of oxalate of lime, and a more moderate display of crystals of the urate of soda, phosphate of ammonia, etc.

Knowing that any verbal description I might attempt would fail to convey an accurate idea of this condition, I have again invoked the assistance of the engraver. Beside the forms given in the cut (and they are but samples of those most constantly appearing,) there are frequently recurring spherical masses of the urates and phosphates, that might be termed microscopic calculi, covering, often, nearly the whole field; and the intervening spaces are everywhere dotted with amorphous urates.

The urine has an acid reaction to litmus paper. This is probably due to an excess of phosphoric acid. Beale says: "It must not be supposed that highly alkaline urine necessarily contains a very large excess of earthy phosphate; for often an excessive quantity of the salts has been found dissolved in acid urine." The urinometer shows the rather unusual specific gravity of 1.030.

Etiologically considered, we may conclude that this disease was probably engendered during the period of gestation; the mother's blood becoming, through her long and forced partial starvation, too impoverished to supply the needed elements to her child in utero for the proper development of its osseous structure.

During the mother's urinary retention, there may have been a degree of uremic poisoning; but that is doubtful. There is no evidence of other causes.

*Pathology.*—The morbid action seems to be a chronic state of osseous inanition; from partial obliteration and diminution of the calibre of the ramifying canals supplying the nutrient cells. This, if long continued, would give the periosteum a greatly increased density, while it would leave the agents of assimilation and nutrition within the cortex, in a perishing condition. I think a dissection of the bone, would show the cortical portion to be a thin, smooth and compact shell, covering a reduced cancellated structure, and an enlarged medullary canal.

Paget, Ashhurst, Gross, Pirrie and others, consider the fragility of old age, and that which may occur in youth, as pathologically identical. They agree that, previous to the osseous fracture, the patient is harassed with severe and fixed pains; that on dissection the bone is shown to be thickened and of roughened outline; while its body, including the periosteum, is a vascular and spongy mass, infiltrated with fat and bloody matter; a form of fatty degeneration.

These are undoubtedly the conditions in the senile variety. And I may add that I have had a corroborative case under my own observation. About nine years ago a cadaver was brought into the dissecting room of the Chicago Medical College, in the long bones of which I counted, if my memory serves me correctly, seven fractures. The body was that of a woman apparently seventy years old, perhaps even older. It presented no indications of constitutional or other disease, unless old age could be so considered. The anterior ridge of the tibiæ was nodulated as in an old case of periostitis. On dissection a good portion of the body of each long bone was found to be an incongruous mass of brittle, spongy tissue, the central canal and periosteum alike obliterated. The cell partitions leaving large interstices, were filled with an oily substance, and the whole was often friable under the fingers.

Several of these bones are now in the collection of the Chicago Medical College Museum.

From this form, the disease under which my patient suffers, differs in many if not in all essential points. She has no premonitory pains, and the bones at all superficial points have a smooth, hard feel, excepting only at the places of fracture; and, when broken, the crepitation seems much the same as would be produced by rubbing together the broken edges of a piece of china ware. It seems also to differ as widely from the fragility of atrophy; this latter disease resulting usually, I believe, from either an inflammatory action engendered by cancer, rheumatism, syphilis, or a structural change in tissues contiguous to the bone; in this last case the fragility being merely local.

The term "Fragilitas Ossium," though common, is undoubtedly an unfortunate one; as indeed is any name that instead of giving some distinct pathological condition, only expresses a result that may have had its origin in any one of a wide range of different causes.

My attendance covers now a period of about four years, during which time the disease has progressed uninterruptedly; and I must say, with a feeling of regret, that I am hopeless of better success in the future; only expecting to be of service in treating the fractures as they may occur.

Since writing the above report, I have been again summoned to this patient, in order to dress a fracture of the left ulna. In this instance the injury resulted from a strain brought to bear upon the bone while the patient was playfully holding to the dress of another child.

## Figures and Tables

**Figure f1:**
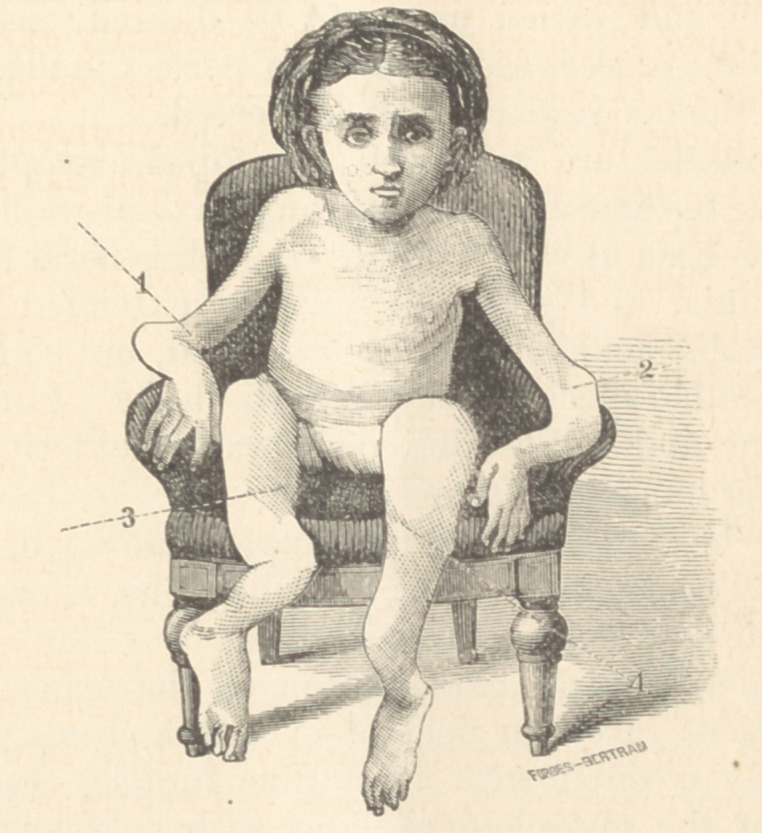


**Figure f2:**