# Erratum

**Published:** 1876-01

**Authors:** 


☞ERRATUM.—In article on "Fragilitas Ossium," page 9, second line from bottom, for "parturition," read *pregnancy*.

